# Diagnosis of schizophrenia with functional connectome data: a graph-based convolutional neural network approach

**DOI:** 10.1186/s12868-021-00682-9

**Published:** 2022-01-17

**Authors:** Kang-Han Oh, Il-Seok Oh, Uyanga Tsogt, Jie Shen, Woo-Sung Kim, Congcong Liu, Nam-In Kang, Keon-Hak Lee, Jing Sui, Sung-Wan Kim, Young-Chul Chung

**Affiliations:** 1 Department of Computer and Software Engineering, Wonkwang University, Iksan, 54538 Korea; 2 Department of Computer Science and Engineering, Jeonbuk National University, Jeonju, Korea; 3 Department of Psychiatry, Jeonbuk National University, Medical School, Geonjiro 20, Jeonju, Korea; 4 Research Institute of Clinical Medicine of Jeonbuk National University-Biomedical Research Institute of Jeonbuk National University Hos Pital, Jeonju, Korea; 5 Department of Psychiatry, Maeumsarang Hospital, Wanju, Jeollabuk-do Korea; 6 Brainnetome Center and National Laboratory of Pattern Recognition, Institute of Automation, Chinese Academy of Sciences, Beijing, 100190 China; 7 University of Chinese Academy of Sciences; CAS Center for Excellence in Brain Science and Intelligence Technology, Chinese Academy of Sciences, Beijing, 100049 China; 8 Department of Psychiatry, Chonnam National University Medical School, Gwangju, Republic of Korea

**Keywords:** Brain network, Functional connectome, Convolutional neural network, Global covariance pooling, Self-attention mechanism, Schizophrenia

## Abstract

Previous deep learning methods have not captured graph or network representations of brain structural or functional connectome data. To address this, we developed the BrainNet-Global Covariance Pooling-Attention Convolutional Neural Network (BrainNet-GA CNN) by incorporating BrainNetCNN and global covariance pooling into the self-attention mechanism. Resting-state functional magnetic resonance imaging data were obtained from 171 patients with schizophrenia spectrum disorders (SSDs) and 161 healthy controls (HCs). We conducted an ablation analysis of the proposed BrainNet-GA CNN and quantitative performance comparisons with competing methods using the nested tenfold cross validation strategy. The performance of our model was compared with competing methods. Discriminative connections were visualized using the gradient-based explanation method and compared with the results obtained using functional connectivity analysis. The BrainNet-GA CNN showed an accuracy of 83.13%, outperforming other competing methods. Among the top 10 discriminative connections, some were associated with the default mode network and auditory network. Interestingly, these regions were also significant in the functional connectivity analysis. Our findings suggest that the proposed BrainNet-GA CNN can classify patients with SSDs and HCs with higher accuracy than other models. Visualization of salient regions provides important clinical information. These results highlight the potential use of the BrainNet-GA CNN in the diagnosis of schizophrenia.

**Supplementary Information:**

The online version contains supplementary material available at 10.1186/s12868-021-00682-9.

## Introduction

Convolutional neural networks (CNNs) are extremely efficient architectures in image and audio recognition tasks. CNNs performed better than other DNNs in the classification of Alzheimer’s disease versus mild cognitive impairment or normal controls [1, 2]. We also previously reported 84.15–84.43% classification accuracies for schizophrenia (SZ) using a 3D CNN model, outperforming support vector machine (SVM) and other 3D CNN models [3]. However, a critical limitation of conventional CNNs is that receptive fields of their filters for feature extraction do not exactly capture graph or network representations of structural or functional connectome data of the brain. Recent research has shown that the representations produced by CNNs can be strengthened by integrating learning mechanisms into the network that help capture graph or network representations between features; one of these models is the BrainNetCNN [4].

The BrainNetCNN, a type of CNN, is composed of novel edge-to-edge (E2E), edge-to-node (E2N), and node-to-graph (N2G) convolutional filters that leverage the topological locality of brain network data. With structural connectome data, the BrainNetCNN framework outperformed a variety of other methods in predicting neurodevelopment [5]. Another model is a global covariance pooling (second-order pooling). Compared with global average pooling (first-order pooling) in existing deep CNNs, global covariance pooling produces covariance matrices deciphering higher order representations with the potential to enhance the nonlinear modeling capacity of deep CNNs [6]. However, a drawback of global covariance pooling is that the second-order pooling block is only applicable at the end of the network. To overcome this, Gao and colleagues proposed a novel network model introducing global second-order pooling across lower to higher layers to exploit holistic image information throughout a network [7]. With the self-attention strategy [8], high-order statistical representations can be trained at every layer, outperforming other methods [6]. Based on current trends, we hypothesized that the BrainNetCNN framework combined with global covariance pooling and self-attention model would achieve a higher performance with functional connectome data. We named this new model the BrainNet-Global covariance pooling-Attention Convolutional Neural Network (BrainNet-GA CNN). The aims of the present study were to perform an ablation study using the BrainNet-GA CNN to analyze resting-state functional magnetic resonance imaging (rsfMRI) data and compare its accuracy in classifying SZ versus healthy controls (HCs) with those of other networks. In addition, we sought to develop an explainable saliency map showing significant connections discriminating between SZ and HCs. These connections were compared to the results of functional connectivity (FC) obtained with univariate analysis.

## Results

### Demographic and clinical characteristics

The diagnoses of patients were SZ (n = 128), schizophreniform disorder (n = 40), and schizoaffective disorder (n = 3). There were no significant differences in age and sex between the SSD and HC groups. However, education was lower in the SSD group compared to the HC group (Table 1).

**Table 1:** Demographic and clinical characteristics of patients with SSDs and HCs

Characteristics	SSDs (n = 171)	HCs (n = 161)	*p*-value (2 Tailed)
Age (years)	34.38 (10.61)	33.73 (10.96)	0.597^b^
Sex			
Male (%)	89 (52.05)	74 (45.96)	0.259^a^
Female (%)	82 (47.95)	87 (54.04)	
Education (years)	13.90 (2.44)	15.26 (2.07)	< 0.001^b^
Duration of illness (months)	77.70 (96.50)	–	–
CDSS Total	5.88 (5.83)	–	–
PANSS			
Positive symptoms	13.69 (8.00)	–	–
Negative symptoms	11.57 (6.55)	–	–
General psychopathology	24.80 (11.35)	–	–
Total score	50.05 (23.26)	–	–
Medication			
Naive/Free (%)	28 (16.37)/29 (16.96)	–	–
Chlorpromazine equivalent (mg/day)	449.33(351.495) (n = 114)	–	–

Data given as mean (SD). ^a^Significant T statistic for the Chi-square test; ^b^Significant T statistic for the independent two sample *t*-test

CDSS, Calgary Depression Scale for Schizophrenia; HCs, Healthy Controls; PANSS, Positive and Negative Syndrome Scale; SSDs, Schizophrenia Spectrum Disorders

### Ablation study on the BrainNet-GA CNN

When two convolutional layers with the Net-GA block were used, the highest accuracy obtained was 83.13%. Regardless of the number of layers, accuracy was consistently better (6–7%) in the network with the Net-GA block compared to the network without the Net-GA block (Table 2). Unexpectedly, performance was the best with one E2E layer (Table 3). As for the hidden units of N2G in the Net-GA block, performance was slightly better with 10 units (Table 4). When the number of output dimensionality of the typical convolutional layer was 64, the best performance was observed. Minimal variations of performance were observed with different numbers of output dimensionality of the E2E layer of the Net-GA block (Table 5).

**Table 2:** Performance comparison by the number of convolutional layers with or without Net-GA block

Layers	Accuracy	Sensitivity	Specificity
1	81.93/75.30	84.21/76.61	79.50/73.91
2	83.13/76.79	85.96/79.65	80.12/73.68
3	83.02/76.81	85.27/79.53	80.88/73.91
4	82.23/75.70	85.38/76.88	78.88/74.53

Data given as with GCP/without GCP (%); Net-GA, Net-Global Covariance Pooling-Attention

**Table 3:** Performance comparison by the number of E2E layers in Net-GA block

The Number of E2E layers	Accuracy	Sensitivity	Specificity
1	83.13	85.96	80.12
2	82.23	85.38	78.88
3	80.72	83.04	78.26

Data given as %

**Table 4:** Performance comparison by the number of the hidden units of N2G in Net-GA block

Number of hidden units	Accuracy	Sensitivity	Specificity
5	82.83	87.13	78.26
10	83.13	85.96	80.12
15	82.53	87.72	77.02
20	82.83	85.96	79.50

Data given as %

**Table 5:** Performance comparison by the number of the output channels

E2E layer	Convolutional layer			
	8	12	16	20
16	80.42	79.95	80.12	80.72
32	81.33	81.93	81.33	82.50
64	82.23	82.83	83.13	83.02
96	81.63	82.23	83.02	82.83
128	81.93	81.33	82.23	82.50

Data given as %

### Performance comparison with other competing methods

**Fig. 1:**
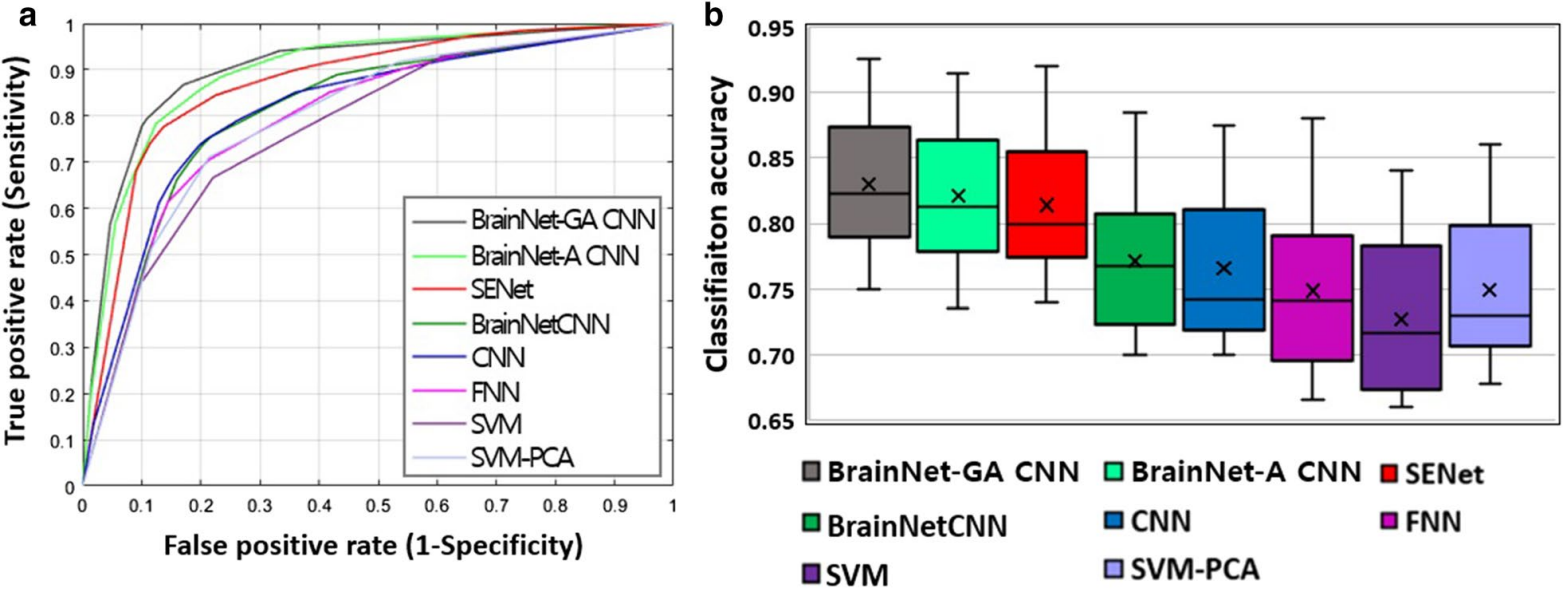
Quantitative performance comparison of the BrainNet-GA CNN with competing methods: **a** ROC curves and **b** box plot graph

The proposed BrainNet-GA CNN showed the best accuracy (83.13%) and area under the curve (89.42%). Its permutation test (10,000 times) was significant (p < 0.001). The next best model was SENet (Table 6 and Fig. 1).

**Table 6:** Performance comparison of the BrainNet-GA CNN with competing methods

	Accuracy	Sensitivity	Specificity	AUC
SVM-PCA	74.90	77.96	71.55	78.85
SVM	72.34	76.91	67.40	76.25
FNNs	74.59	77.72	71.25	78.82
CNNs	76.79	79.65	73.68	80.69
BrainNetCNNs	77.04	78.98	75.00	81.74
SENet	81.21	83.38	79.10	86.85
BrainNet-A CNN	82.04	84.47	79.63	88.41
BrainNet-GA CNN	83.13	85.96	80.12	89.42

Data given as %, AUC, Area under the curve; BrainNet-A CNN, BrainNet-Attention CNN; BrainNet-GA CNN, BrainNet-Global Covariance Pooling-Attention CNN; CNNs, Convolutional Neural Networks; FNNs, Fully Connected Neural Networks; PCA, Principal Component Analysis; SENet, Squeeze and Excitation Network; SVM, Support Vector Machine

### Discriminative connections

**Fig. 2:**
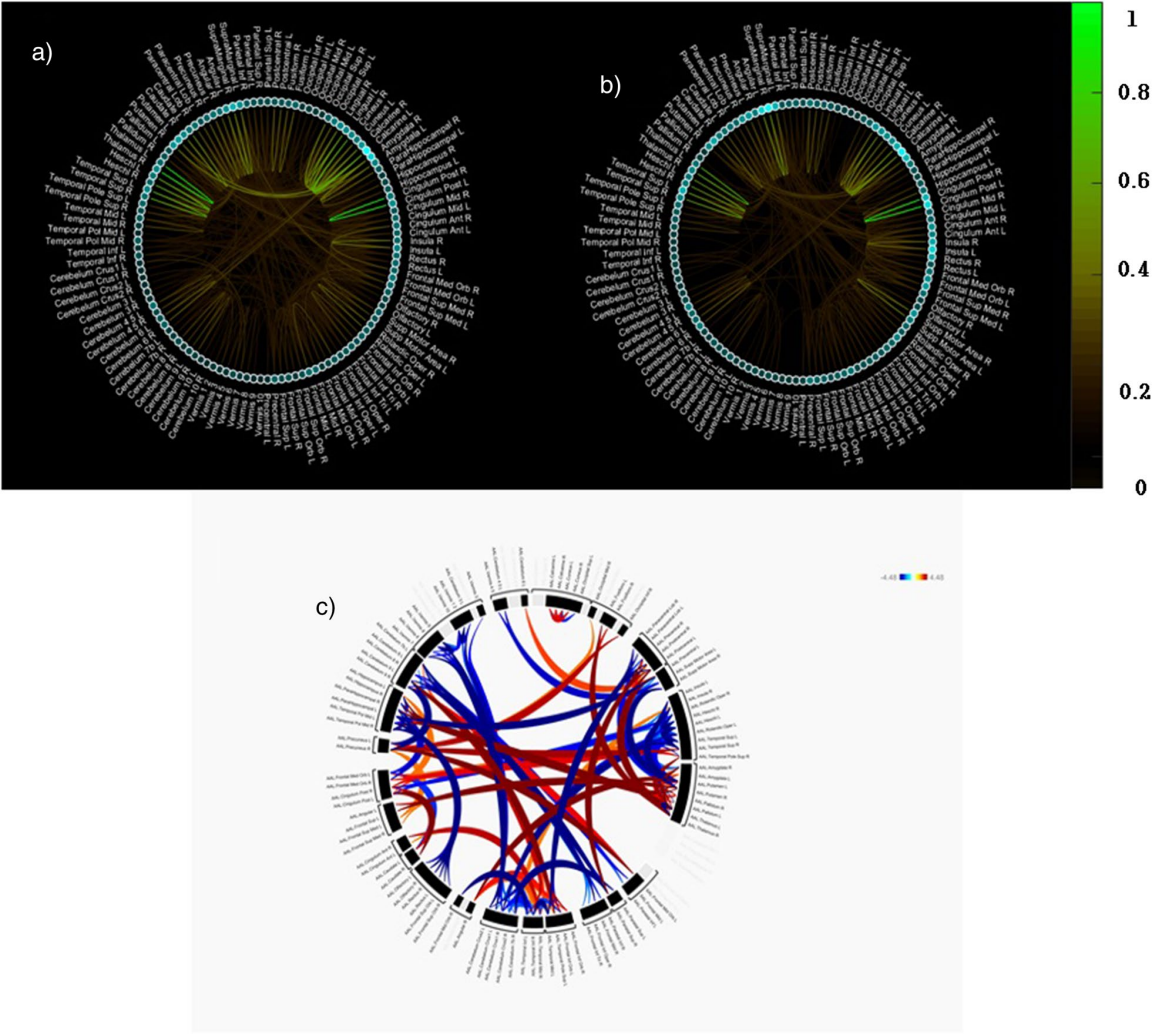
^**†**^Discriminative connections between brain regions for the classification of SSDs vs. HCs: **a** results of partial derivatives on a target class of SSDs and **b** HCs, and **c** ^§^Circular plot showing increased (red color) or decreased (blue color) functional connectivity in patients compared to controls. ^†^Green line and bar represent connectivity strength. The brighter color is, the greater its importance in the classification; Small circle in sky blue represents nodal strength. The more circle is filled, the greater its importance in the classification; ^§^Red and blue lines represent hyperconnectivity and hypoconnectivity, respectively and darker line means more higher value

Regarding the connectivity strength between nodes, the top 10 discriminative connections were between the brain regions of the left posterior cingulate gyrus and right posterior cingulate gyrus; left thalamus and right thalamus; left calcarine sulcus and right cuneus; and left Heschl’s gyrus and right Heschl’s gyrus. The brain regions with highest nodal strength were the left calcarine sulcus, right amygdala, left putamen, right thalamus, and right supramarginal gyrus (Table 7 and Fig. 2).

**Table 7:** Top 10 discriminative connections

	Connectivity strength	Nodal strength
1	Left posterior cingulate gyrus—right posterior cingulate gyrus (1)	Left calcarine sulcus
2	Right thalamus—left thalamus (1)	Right amygdala
3	Right cuneus—left calcarine sulcus (0.71)	Left putamen
4	Right superior temporal gyrus—left superior temporal gyrus (0.69)	Right thalamus
5	Right Heschl’s gyrus—left Heschl’s gyrus (0.69)	Right supramarginal gyrus
6	Left lingual gyrus—left calcarine sulcus (0.67)	Right putamen
7	Right cuneus—right calcarine sulcus (0.59)	Right caudate nucleus
8	Right caudate nucleus—left caudate nucleus (0.57)	Right calcarine sulcus
9	Left lingual gyrus—right lingual gyrus (0.56)	Left posterior cingulate gyrus
10	Right supramarginal gyrus—left angular gyrus (0.55)	Left angular gyrus

### Functional connectivity analysis

Compared to the HC group, the SSD group exhibited significantly increased FC between the brain regions of the cingulate gyrus and inferior frontal gyrus; left superior frontal gyrus and right inferior frontal gyrus; left angular gyrus and left inferior frontal gyrus; and right cuneus and left calcarine sulcus. Additionally, the SSD group showed decreased FC between the brain regions of the putamen and insular cortex, left Heschl’s gyrus and right Heschl’s gyrus and left superior temporal gyrus and, right superior temporal gyrus compared to the HC group (Table 8). Partial correlation analysis revealed significant positive relationships between the connectivity of the left anterior cingulate gyrus and left triangularis inferior frontal gyrus and positive symptom subscale, connectivity of the right anterior cingulate gyrus and left orbital inferior frontal gyrus and negative symptom subscale and connectivity of the right cuneus and left calcarine and positive symptom subscale, general psychopathology subscale and, total score of the PANSS (Additional file 1: Table S1).

**Table 8:** Aberrant functional connections in patients with schizophrenia spectrum disorders

Brain region	*t* value	Effect size	*p*-unc	*p*- FDR	Brain region
SSDs > HCs					
Left posterior cingulate gyrus	6.38	0.150	< 0.001	< 0.001	Left orbital inferior frontal gyrus
	5.17	0.130	< 0.001	< 0.001	Right orbital inferior frontal gyrus
	4.35	0.110	< 0.001	0.005	Left triangularis inferior frontal gyrus
Right posterior cingulate gyrus	6.33	0.140	< 0.001	< 0.001	Left orbital inferior frontal gyrus
	5.20	0.130	< 0.001	< 0.001	Right orbital inferior frontal gyrus
	4.24	0.099	< 0.001	0.007	Left triangularis inferior frontal gyrus
Left orbito medial frontal gyrus	4.72	0.130	< 0.001	0.002	Right orbital inferior frontal gyrus
	4.70	0.110	< 0.001	0.002	Right operculum inferior frontal gyrus
	4.24	0.120	< 0.001	0.007	Left orbital inferior frontal gyrus
Right orbito medial frontal gyrus	4.04	0.097	< 0.001	0.001	Left operculum inferior frontal gyrus
	3.82	0.090	< 0.001	0.001	Left triangularis inferior frontal gyrus
	3.40	0.081	< 0.001	0.001	Right triangularis inferior frontal gyrus
Left anterior cingulate gyrus	4.21	0.110	< 0.001	0.001	Left triangularis inferior frontal gyrus
	3.83	0.100	< 0.001	0.001	Right triangularis inferior frontal gyrus
	3.82	0.093	< 0.001	0.001	Left operculum inferior frontal gyrus
Right anterior cingulate gyrus	4.42	0.110	< 0.001	0.004	Left orbital inferior frontal gyrus
	3.78	0.094	< 0.001	0.001	Left triangularis inferior frontal gyrus
	3.48	0.091	< 0.001	0.001	Right triangularis inferior frontal gyrus
Left superior frontal gyrus	4.54	0.110	< 0.001	0.003	Right operculum inferior frontal gyrus
	4.37	0.110	< 0.001	0.005	Right triangularis inferior frontal gyrus
Left precuneus	4.19	0.100	< 0.001	0.008	Left orbital inferior frontal gyrus
Left angular gyrus	4.24	0.120	< 0.001	0.007	Left triangularis inferior frontal gyrus
Right cuneus	4.17	0.130	< 0.001	0.008	Left calcarine sulcus
Left calcarine sulcus	5.40	0.140	< 0.001	< 0.001	Left cerebellum 6
Left middle cingulate gyrus	4.73	0.110	< 0.001	0.002	Left triangularis inferior frontal gyrus
SSDs < HCs					
Left putamen	−5.20	−0.120	< 0.001	< 0.001	Right insular cortex
Right putamen	−5.94	−0.140	< 0.001	< 0.001	Right insular cortex
	−5.20	−0.110	< 0.001	< 0.001	Left insular cortex
Left Heschl’s gyrus	−6.38	−0.160	< 0.001	< 0.001	Right Heschl’s gyrus
	−4.45	−0.110	< 0.001	0.004	Right superior temporal gyrus
Left superior temporal gyrus	−4.80	−0.140	< 0.001	0.001	Right superior temporal gyrus

Whole-brain thresholded at FDR corrected p < 0.01, FDR, False Discovery Rate; HCs, Healthy Controls; SSDs, Schizophrenia spectrum disorders

## Discussion

To overcome the limitation of previous deep learning (DL) methods not capturing graph or network representations of connectome data, we developed the BrainNet-GA CNN by incorporating BrainNetCNN and global covariance pooling into the self-attention mechanism. Advancement of scientific knowledge is described below in terms of methodological and clinical aspects.

### Methodological aspects

In the ablation study, favorable performance was reported in the network architecture composed of only two convolutional layers with Net-GA blocks. Relevant studies [8, 9] have shown that deep convolutional networks with covariance pooling outperformed other competing methods in a large-scale visual recognition task. Unlike the results of the BrainNetCNN study [5], there was no benefit when stacking multiple E2E layers for our classification task. It seems that the features transformed by multiple E2E layers have a negative effect on extracting higher order features of the next E2N layer, which was not used in their original study [5]. Although ten units performed slightly better, the overall difference was small.

The best accuracy was obtained with the BrainNet-GA CNN, compared with other competing methods. Two characteristics of our model may have contributed to this superiority. First, because second-order pooling in the E2N layer captures higher order representations, this may lead to more discriminative features. The covariance matrix produced by second-order pooling is known to improve representation power by quadratic modeling. The *i*-th row can be interpreted to indicate statistical dependency between the *i*-th brain region and all other brain regions. We believe that this technique is a proper approach for neuroimaging data in which correlations or network features between brain regions are crucial. This may be partially supported by the finding that performance dropped a little when second-order pooling was not used in our model. Second, adopting the self-attention mechanism may enhance classification performance by effectively learning graph-wise high-order representations at every convolutional layer and recalibrating filter responses. Because the self-attention mechanism allows covariance pooling to be conveniently plugged into any location of the convolutional layers, it helps build deep CNNs. Unlike the BrainNetCNN, our model is flexible and has a deep structure. This may have contributed to the capture of richer statistics of deep features and improvement of the representation and generalization abilities of deep CNNs. Although implementation of a typical convolutional layer before the Net-GA block was necessary to apply the self-attention mechanism, it may be criticized that features extracted from this convolutional layer may not contain graph or network representations of our connectome data. However, we regarded this convolutional layer as a local sparse feature extractor since a typical convolution operation with small kernels does not significantly distort the topological characteristic of the correlation matrix.

### Clinical implications

Using multivariate DL techniques, neuroimaging-based single-subject prediction of psychiatric disorders has gained increasing attention in recent years. Several studies have employed DL methods to classify SZ and HCs. Using sMRI data, two studies applied a DBN to the original pre-processed images and obtained accuracy rates of 91% and 73.6%, respectively [10, 11]. Applying SAE with weight sparsity control to rsfMRI data, classification of SZ vs. and controls with an accuracy of 85.5% was reported [12]. Other studies with rsfMRI data reported accuracy rates of 79–92% in SZ using autoencoder-based two- or three-stage architecture [13–15]. FC analysis with rsfMRI data produces a correlation matrix representing inter-correlation between voxels or regions. However, previous DL methods used in the classification of SZ and HCs did not capture inter-correlation features of the brain network. For example, DNN with weight sparsity control requires a 1D input feature vector, thereby losing spatial information between voxels or regions. Although the CNN used in our previous work [3] can preserve spatial locality with the use of 3D data, this model did not capture topological locality of the brain network. The accuracy of our model, BrainNet-GA CNN, was the best in the classification of SSDs and HCs, outperforming BrainNetCNN by 6.09%. This suggests our proposed model is an optimally designed approach to capture inter-correlation features of functional connectome data.

Using the gradient-based explanation method, we identified discriminative functional connections between the brain regions contributing significantly to the recognition of SSDs. Among the top 10 discriminative connections, some regions (posterior cingulate gyrus and angular gyrus) were related to the DMN and others to the auditory network (superior temporal gyrus and Heschl’s gyrus) and thalamus network. The DMN is involved in complex self-referential stimuli, such as mental time travel, perspective taking, and theory of mind [16]. Accumulating evidence suggests that the DMN is abnormal in SZ, although the results have been mixed [8]. It is of interest that the best discriminative connectivity was an interhemispheric connection in the posterior cingulate gyrus. The posterior cingulate cortex (PCC), a key node in the DMN, has a central role in supporting internally-directed cognition showing increased activity when individuals retrieve autobiographical memories or plan for the future [17]. Increases and decreases of resting FC around the PCC have been reported in both patients and their first-degree relatives [18, 19]. In SZ, the auditory cortex is closely associated with auditory verbal hallucinations, which have been proposed to be a result of abnormally elevated resting-state activity in the auditory cortex or from the DMN [20]. The second most discriminative connection was an interhemispheric connection in the thalamus. The thalamus represents an essential hub for cognitive processes and an interface between sensory and motor systems. Brain-wide analysis of FC in SZ revealed that thalamic-related aberrant connectivities were prominent at the chronic stage of SZ [21]. In the task-based fMRI data, the most-significant, stable and discriminative FC changes involved increased correlations between thalamus and other cortical regions [22]. Interestingly, some of these regions (cingulate gyrus, superior temporal gyrus, Heschl’s gyrus and cuneus) were also found to be significant in the FC analysis.

Overall pattern of the FC analysis was a more widespread occurrence of increased connectivities in patients with SZ compared with HCs. This seems in contrast with the results of previous studies that global/average connectivity strength was significantly reduced in SZ compared to controls [23, 24]. However, it should be noted that there are many other studies reporting increased connectivities in the resting-state DMN [25], thalamo-sensorimotor link [26] and computational modeling [27]. The most prominent aberrant connections in SZ were between the cingulate and inferior frontal gyri. Our findings are partially supported by the results of Li et al. study [21]. That in patients with first episode SZ, 90% of the FC changes involved the frontal lobes, mostly the inferior frontal gyrus whereas the PCC was one of the areas showing the most prominent changes in chronic SZ. Interestingly, we found positive relationship between the connectivity strength of anterior cingulate gyrus with inferior frontal gyrus and positive or negative symptoms. The anterior cingulate cortex (ACC) is known to be involved in the affect regulation, conflict monitoring and executive control of cognition [28]. The inferior frontal gyrus is involved in attention control and response inhibition [29]. Therefore, it may be speculated that aberrant connectivity in the ACC and inferior frontal gyrus affects their functioning which may in turn lead to development of positive or negative symptoms in SZ. We observed decreased connectivities in the Heschl’s gyrus and superior temporal gyrus. Similarly, Venkataraman and colleagues reported decreased connectivity between the temporal cortices bilaterally in SZ [30]. However, no significant correlation was found between these hypoconnectivity and psychopathology. Lastly, increased connectivity between the cuneus and calcarine sulcus was shown in individuals with SZ compared to HCs. The cuneus (Brodmann area 17) receives visual information from the same-sided superior quadrantic retina and is primarily involved in basic visual processing. The calcarine fissure is a deep sulcus located on the medial surface of the occipital lobe. Multiple lines of evidence indicate that there are reduced intrinsic visual cortical connectivity [31] and decreased connection in high-visual network which was found to be correlated with the severity of positive symptoms in SZ [32]. Thus, our findings on the connectivity plus its correlation with psychopathology suggest that impaired visual networks may also contribute to the development of psychopathology in SZ. On the other hand, while medial, superior, and inferior frontal gyri were found to be significant in the FC analysis, these regions were not identified as such in the gradient-based explanation method. In addition, bilateral connections between the same regions were highly prominent in the gradient-based explanation method, whereas unilateral or bilateral connections between different regions were more common in the FC analysis. These discrepancies may be due to the different methodologies used in the two analyses.

This study has several limitations. First, because the number of subjects used for the training and test phases was small, it is unclear how well these findings will generalize to different samples. Validation experiments will also be necessary if the transfer classification model is applied to a clinical population at a new imaging site. Second, although the proportion of patients with an antipsychotic naïve or free state was approximately 33%, most of the patients were on medication at the time of scanning. Antipsychotics are known to affect FC [33], this factor should be controlled if possible. Despite these caveats, this is the first study to apply a graphical approach based on the CNN to functional connectome data in SSDs. Overall, the BrainNet-GA CNN showed high accuracy in the classification of SSDs and HCs, outperforming other competing methods. Some of the discriminative connections were associated with DMN and auditory network brain regions. Furthermore, some of the discriminative connections identified by DL and conventional univariate methods were similar. These results highlight a potential use of the BrainNet-GA CNN in the diagnosis of SZ.

## Methods

### Participants

All participating patients (n = 171) met DSM-IV-TR criteria for schizophrenia spectrum disorders (SSDs) according to the Structured Clinical Interview for DSM-IV (SCID) [34, 35]. Individuals with alcohol- or drug-use disorders within the past 6 months, intellectual disability (IQ ≤ 70), current or historical neurological disorders, pregnancy, and claustrophobia were excluded from the study. HCs were required to have no previous or current psychiatric disorders, neurological disorders, or significant medical conditions.

## Declaration

### Clinical assessment

The severity of symptoms was evaluated within a week of fMRI scanning using the positive and negative syndrome scale [36] and, the Calgary Depression Scale for Schizophrenia^7^. The PANSS and CDSS were administered by trained psychiatrists.

### Image acquisition and preprocessing

Resting-state functional and structural MRI (rsfMRI and sMRI) data were obtained at the Jeonbuk National University Hospital on a 3 T Verio scanner (Siemens Magnetom Verio, Erlangen, Germany) using a 12-channel standard quadrature head coil. We collected a 5-min resting-state scan consisting of 150 contiguous echo-planar imaging functional images (TR: 2000 ms; TE: 30 ms; flip angle: 90°; FOV: 240 mm; image matrix: 64 × 64 mm; voxel size = 1.0 × 1.0 × 1.0 mm [3]; 176 slices). MRI data preprocessing was conducted in a standard way using the Statistical Parametric Mapping software package, ver 12. The criteria for excessive head motion were translation > 2 mm or rotation > 2° in any direction. Participants for whom more than 10% of volumes showed excessive head motion were excluded from the analysis. The linear trend was removed through the time course, and the band-pass filter (0.008 < f < 0.09 Hz) was applied.

### Functional connectivity analysis

Time series of the voxels within the ROI were averaged to generate the regional time series for the automated anatomical labeling (AAL) atlas. The FC matrix was computed by correlating time series data between every pair in the AAL atlas using the CONN toolbox. Group comparison was performed using ANCOVA with education as covariate. For the contrast map, we applied the cluster-level extent threshold of p < 0.01, which was corrected for multiple comparisons using the false discovery rate (Additional file 2). Partial correlations were carried out controlling for age, sex, education, duration of illness, chlorpromazine equivalent doses and head motion (framewise displacement) on the relationship between the rsFC z-values of brain regions showing significant between- group differences and PANSS. The significance level was set at a cluster-level of *p* < 0.05, and data were not corrected for multiple comparison because of the exploratory nature of the evaluation.

### Net-global covariance pooling-attention block

**Fig. 3:**
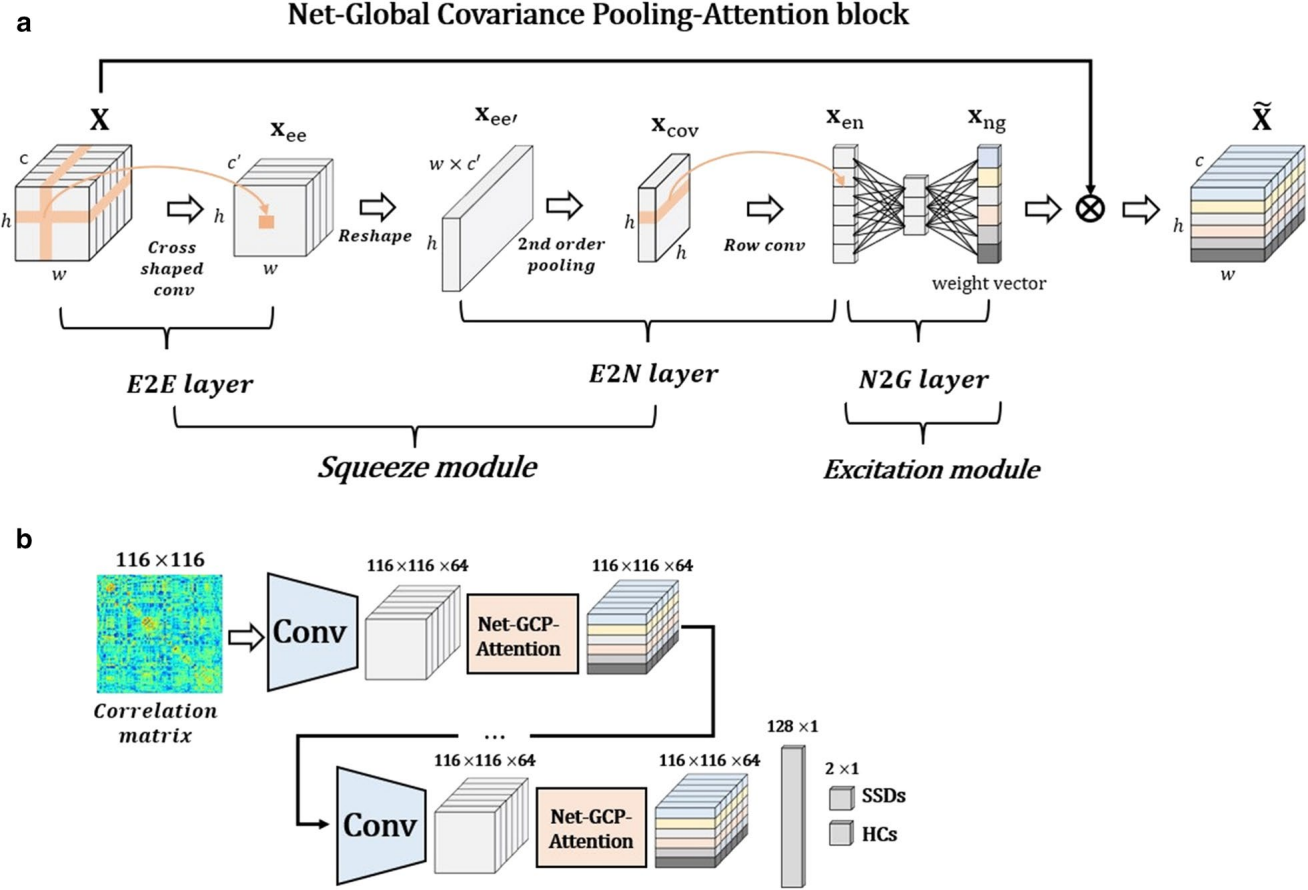
**a **Overview of Net-Global Covariance Pooling-Attention (Net-GA) block and **b** architecture of the BrainNet-GA CNN for the classification of SSDs vs. HCs. The Net-GA block consists of the BrainNetCNN (E2E, E2N and N2G filters) combined with global covariance pooling (2nd order pooling) and squeeze-excitation network (attention model). The BrainNet-GA CNN consists of typical convolutional layer plus Net-GA block

The brain FC can be expressed as the complete graph $G = \left( {E,{ }B} \right),$ where $B$ is a set of nodes reflecting 116 brain regions and $E$ is a weighted adjacency matrix of edges. To capture graph representations of a functional connectome, we adopted graph-wise convolutional filters in the BrainNetCNN [5], which were composed of E2E, E2N, and N2G. However, the block was modified by applying two more methods, i.e., second-order pooling and squeeze-excitation network as a self-attention model, and was named the Net-Global Covariance Pooling-Attention (Net-GA) block (Fig. 3).

Unlike the BrainNetCNN [5] second-order pooling was inserted before the row convolutional filter in the E2N layer. To this end, the 3D output tensor ${\mathbf{x}}_{{{\text{ee}}}} \in {\mathbb{R}}^{{h \times w \times c^{\prime}}}$ of the E2E layer was reshaped to the two-dimensional matrix ${\mathbf{F}}_{{{\text{re}}}} :{\mathbf{x}}_{{{\text{ee}}}} \to {\mathbf{x}}_{{{\mathbf{ee^{\prime}}}}} \in {\mathbb{R}}^{h \times M}$ where the *i*-th row indicates the representations of *i*-th brain regions. Given the matrix ${\mathbf{X}}_{\text{ee}^\prime}$ consisting of M-samples and h-dimensional features, the sample covariance matrix of ${\mathbf{X}}_{\text{ee}^\prime}$ can be written as follows:

1 $$ {\mathbf{F}}_{{\text{cov}}} :{\mathbf{x}}_{\text{ee}^\prime} \to {\mathbf{x}}_{{\text{cov}}} = {\mathbf{x}}_{\text{ee}^\prime} {\mathbf{A}}{\mathbf{x}}_{\text{ee}^\prime}^{{\text{T}}} ,{\mathbf{A = }}\frac{1}{M}\left( {{\mathbf{I}} - \frac{1}{M}{\mathbf{JJ}}^{{\text{T}}} } \right) $$

where ${\mathbf{I}}$ is the ${ }M \times M$. identity matrix, ${\mathbf{J}}$ represents the $M$-dimensional vector, which is composed of one, and ${\text{T}}$ denotes the matrix transpose. We performed a row-wise group convolutional filter by considering each row as a group, ${\mathbf{F}}_{{{\text{rconv}}}} :{\mathbf{x}}_{{{\text{cov}}}} \to {\mathbf{x}}_{{{\text{en}}}} \in {\mathbb{R}}^{h \times 1}$, to maintain characteristics of the functional connectome data. Through the proposed E2N layer, the input tensor from the E2E layer was transformed into region-wise sparse representations corresponding to the number of brain regions, which can be defined as follows:

2 $$ {\mathbf{F}}_{{{\text{E}}2{\text{N}}}} = {\mathbf{F}}_{{{\text{rconv}}}} \circ {\mathbf{F}}_{{{\text{cov}}}} \circ {\mathbf{F}}_{{{\text{re}}}} :{\mathbf{x}}_{{{\text{ee}}}} \to {\mathbf{x}}_{{{\text{en}}}} \in {\mathbb{R}}^{h \times 1} $$

The Net-GA block is a computational module that can build the enhanced tensor ${\tilde{\mathbf{X}}} \in {\mathbb{R}}^{h \times w \times c}$ from its original tensor ${\mathbf{X}} \in {\mathbb{R}}^{h \times w \times c}$, which can be defined as foows:

3 $$ {\mathbf{F}}_{{{\text{GBCP}}}} = {\mathbf{F}}_{{{\text{EX}}}} \circ {\mathbf{F}}_{{{\text{SE}}}} :{\mathbf{X}}{ } \to {\tilde{\mathbf{X}}} $$

where ${ }{\mathbf{F}}_{{{\text{SE}}}} = {\mathbf{F}}_{{{\text{E}}2{\text{N}}}} \circ {\mathbf{F}}_{{{\text{E}}2{\text{E}}}}$ is the squeeze function and ${ }{\mathbf{F}}_{{{\text{EX}}}} = { }{\mathbf{F}}_{{{\text{N}}2{\text{G}}}}$ denotes the excitation function. For the squeeze operation, an input tensor was fed to the E2E layer to encode the edge strengths over a pair of brain regions. To decrease the computational cost of second-order pooling at the following layer, we also reduced the number of channels from $c$ to $c^{\prime}$, and the E2E layer of the proposed squeeze operation is defined as follows:

4 $$ {\mathbf{F}}_{{{\text{E}}2{\text{E}}}} :{\mathbf{X}} \to {\mathbf{x}}_{{{\text{ee}}}} \in {\mathbb{R}}^{{h \times w \times c^{\prime}}} $$

For the excitation operation, we employed the N2G layer. This aims to summarize the responses of all brain regions into a single response. In the N2G layer, the dimensionality of input vector ${\mathbf{x}}_{{{\text{en}}}}$ was decreased by passing the bottleneck layer with a reduced ratio. We then increased the vector from the reduced size to its original size, and activated the output vector using the sigmoid function, ${\mathbf{F}}_{{{\text{fc}}}} :{ }{\mathbf{x}}_{{{\text{en}}}} \to {\mathbf{x}}_{{{\text{ng}}}} \in {\mathbb{R}}^{h \times 1}$. The final enhanced tensor ${\tilde{\mathbf{X}}}$ computed by the proposed excitation operation can be obtained by

5 $$ {\mathbf{F}}_{{{\text{N}}2{\text{G}}}} = {\mathbf{F}}_{{{\text{rmul}}}} \user2{ }:{\mathbf{X}},\user2{ } {\mathbf{x}}_{{{\text{ng}}}} \to {\tilde{\mathbf{X}}} \in {\mathbb{R}}^{h \times w \times c} \user2{ } $$

where ${ }{\mathbf{F}}_{{{\text{rmul}}}}$ denotes a row-wise multiplication between the input tensor ${\mathbf{X}} = \left[ {{\mathbf{x}}_{1} ,{\mathbf{x}}_{2} , \ldots ,{\mathbf{x}}_{h} \in {\mathbb{R}}^{w \times c} } \right]$ and the weight vector ${ }{\mathbf{x}}_{{{\text{ng}}}} = \left[ {a_{1} ,a_{2} , \ldots ,a_{h} } \right]$. The output tensor ${\tilde{\mathbf{X}}}$ was highlighted, helping to boost representation discriminability.

### BrainNet-GA CNN architecture

The BrainNet-GA CNN consists of a typical convolutional layer, Net-GA block, and fully connected classification layer (Fig. 3). For a detailed description, see the Additional file 1.

## Experiments

We conducted an ablation analysis on the proposed BrainNet-GA CNN and quantitative performance comparisons with competing methods using the nested tenfold cross validation strategy. To avoid possible bias caused by the random dataset partitioning, the cross-validation was repeated 10 times independently, and the average score was reported. Hyperparameters such as varying regularization factors, weight decay, and network architecture, were empirically tuned and optimized. We optimized two important hyperparameters, an initial learning rate and a weighting factor of L1 regularization, using Bayesian optimization. The performance was evaluated using accuracy, sensitivity, and specificity. Also, we plotted the receiver operating characteristic curve of BrainNet-GA CNN and other competing methods, including SVM, fully connected neural network, CNN, squeeze and excitation network (SENet) [8], and BrainNetCNN [5]. For a detailed description, see the Additional file 1.

### Discriminative connections

To discover discriminative functional connections between the brain regions that make significant contributions to the recognition of SDDs, we used the gradient-based explanation method [37]. To obtain an explainable saliency map, after choosing a target class (SSDs or HCs), we fed validation data to the explanation method, and entire saliency maps were linearly integrated and normalized. Connectivity strength between the nodes and nodal strength (sum of edge weights attached to a node within a network) were estimated.

## Supplementary Information

**Additional file 1.** Implementation of competing methods.

**Additional file 2.** Contrast maps.

## Data Availability

All the data presented and analyzed in this study are fully available from the authors upon request.
