# Correction to: Diagnosis of schizophrenia with functional connectome data: a graph-based convolutional neural network approach

**DOI:** 10.1186/s12868-022-00700-4

**Published:** 2022-03-14

**Authors:** Kang-Han Oh, Il-Seok Oh, Uyanga Tsogt, Jie Shen, Woo-Sung Kim, Congcong Liu, Nam-In Kang, Keon-Hak Lee, Jing Sui, Sung-Wan Kim, Young-Chul Chung

**Affiliations:** 1 Department of Computer and Software Engineering, Wonkwang University, Iksan, 54538 Korea; 2 Department of Computer Science and Engineering, Jeonbuk National University, Jeonju, Korea; 3 Department of Psychiatry, Jeonbuk National University, Medical School, Geonjiro 20, Jeonju, Korea; 4 Research Institute of Clinical Medicine of Jeonbuk National University-Biomedical Research Institute of Jeon- buk National University Hospital, Jeonju, Korea; 5 Department of Psychiatry, Maeumsarang Hospital, Wanju, Jeollabuk-do Korea; 6 Brainnetome Center and National Laboratory of Pattern Recognition, Institute of Automation, Chinese Academy of Sciences, Beijing, 100190 China; 7 University of Chinese Academy of Sciences; CAS Center for Excellence in Brain Science and Intelligence Technology, Chinese Academy of Sciences, Beijing, 100049 China; 8 Department of Psychiatry, Chonnam National University Medical School, Gwangju, Republic of Korea

## Correction to: BMC Neuroscience (2022) 23:5 https://doi.org/10.1186/s12868-021-00682-9

Following publication of the original article [1], it was reported an incorrect affiliation was assigned to Il-Seok Oh.

Il-Seok Oh was incorrectly assigned affiliation 1. The correct affiliation is: Department of Computer Science and Engineering, Jeonbuk National University, Jeonju, Korea.

The original article [1] has been updated.
