# Supplementary material for: Diagnosis of schizophrenia with functional connectome data: a graph-based convolutional neural network approach
Source: BMC Neurosci. 2022 Jan 17;23:5. doi: 10.1186/s12868-021-00682-9 (PMC8764800; doi:10.1186/s12868-021-00682-9)
Supplement: Supplementary file 1 — Additional file 1. Implementation of competing methods. [file 12868_2021_682_MOESM1_ESM.docx]

**Diagnosis of schizophrenia with functional connectome data: a graph-based convolutional neural network approach**

**Kang-Han Oh^1^, Il-Seok Oh^1^, Uyanga Tsogt^2^, Jie Shen^2^, Woo-Sung Kim^2,3^, Congcong Liu^2^, Nam-In Kang^4^, Keon-Hak Lee^4^, Jing Sui^5,6^, Sung-Wan Kim^7^, Young-Chul Chung^2,3^**

**Supplement**

**Implementation of the BrainNet-GA CNN**

Each proposed convolutional layer with 64 feature maps was comprised of 3 × 3 convolutional filter, leaky rectified linear, batch normalization and Net-GA block sequentially. After the convolutional layers, we used fully connected (FC) layer with 128 × 1 and 2 × 1 nodes and final output was activated by the softmax function. The high-level abstracted features were typically interpreted in the FC layer. We did not use the scale pooling function to maintain network representations of the functional connectome data.

For the Net-GA block, we employed leaky rectified linear for the activation function of every layer except last part of N2G layer activated by sigmoid function. For E2E layer, dimensionality of the tensor’s channel was reduced from 64 to 16. Unlike E2E layer for which the tensor dimensionality was preserved, E2N transformed 116 × 116 matrix into 116 × 1 matrix and was mounted before N2G layer and after E2E layer. Similarly, N2G layer was used after E2G layer due to its properties and mechanism. With regard to the second-order pooling, size of the sample covariance matrix was fixed as 116 × 116 because we did not alter the connectome data scale at every layer. In order to decrease the computational complexity of sample covariance matrix, we reduced number of output channels from 64 to 16 in E2E layer. For N2G layer including FC layer, we empirically set number of units in the hidden layer to ten.

For the optimization, we employed the Stochastic Gradient Descent (SGD) with a mini-batch size of ten, a momentum of 0.9, an initial learning late of $1\times{10}^{-6}\sim1\times{10}^{-8}$, and a weight decay of $1\times{10}^{-6}$. Since the most cases reached a convergence state within 150 epochs, we set the final number of epochs to 150. For the cost function, the cross-entropy loss was used to minimize the error between the predicted and real outcome. To decrease the influence of less discriminative features, we applied Lasso (L1) regularization using the weighting factor between $1\times{10}^{-4}\sim1\times{10}^{-6}$.

**Implementation of competing methods**

For the classical machine learning method, we employed the support vector machine (SVM) with nonlinear kernel. The experiments were conducted in two ways where raw features without any preprocessing and principal component analysis (PCA) based sparse features were fed to SVM. In the first case, connectome data of 116 × 116 was reshaped to 6670 × 1 vector reflecting upper triangular values of the symmetric data. In the second case, we extracted reduced features of size 40 × 1 by applying PCA to original raw features of size 6670 × 1. Note that we empirically set the reduction size to 40, which achieved the best classification accuracy. The deep learning-based competing approaches consisted of fully connected neural network (FNN), convolutional neural network (CNN), squeeze and excitation network (SENet)^1^ and BrainNetCNN [2] Similar to the SVM, 1D vector of size 6670 × 1 was used for the input of FNNs. The architecture was composed of input layer of size 6670 × 1, two hidden layers of size 256 × 1, 128 × 1 and output of size 2 × 1. The CNN approach had a same architecture with the proposed network without Net-GA block. Similarly, only difference between the SENet and BrainNet-GA CNN was that self-attention module was mounted after typical convolutional layer in the BrainNet-GA CNN. The SE module was composed of the channel wise global average pooling and FC layer. We set a reduction ratio of hidden layer to six where the best accuracy was reported in the relevant study [1]. The BrainNetCNN architecture consisted of E2E, E2N, N2G and FC layers. Kawahara et al mentioned that multiple E2E layers can be consecutively stacked before conducting E2N layer, yielding the best accuracy with two stacked E2E layers [2]. However, in our experiment, negative effect was observed when using multiple E2E layers and a same phenomenon also occurred in the experiment using E2E layer of the Net-GA block. We thus employed a single E2E as the first layer and its architecture consisted of E2E with 64 channels, E2N with 128 channels and N2G with 128 nodes. For every DNN model, we applied regularization techniques such as dropout of ratio 0.25 before the last layer, L1 penalty of weighting factor $1\times{10}^{-3}\sim1\times{10}^{-6}$; and the loss function is the cross-entropy. As the optimizer, SGD with a mini-batch size of ten, an initial learning late of $1\times{10}^{-6}\sim1\times{10}^{-9}$, a momentum of 0.9 and a weight decay of $1\times{10}^{-6}$ was used. As the activation function, LeakyReLU was used for the last of every layer except the output layer with the softmax function.

**table S1. Correlation analysis results between the PANSS score and Z score of the regions with significant differences between groups**

| Connectivity | *r* value | *p* value |
| --- | --- | --- |
| Positive symptoms total |  |  |
| Right posterior cingulate gyrus – Left triangularis inferior frontal gyrus | -0.077 | 0.497 |
| Left orbito middle frontal gyrus – Right orbito inferior frontal gyrus | 0.151 | 0.179 |
| Left orbito middle frontal gyrus – Right operculum inferior frontal gyrus | 0.146 | 0.194 |
| Left orbito middle frontal gyrus – Left orbito inferior frontal gyrus | 0.184 | 0.100 |
| Right orbito middle frontal gyrus – Right triangularis inferior frontal gyrus | -0.014 | 0.903 |
| Right orbito middle frontal gyrus – Left triangularis inferior frontal gyrus | 0.110 | 0.329 |
| Left anterior cingulate gyrus – Right triangularis inferior frontal gyrus | 0.187 | 0.094 |
| Left anterior cingulate gyrus – Left triangularis inferior frontal gyrus | 0.232 | 0.037 |
| Left anterior cingulate gyrus – Left operculum inferior frontal gyrus | 0.134 | 0.233 |
| Right anterior cingulate gyrus – Left orbito inferior frontal gyrus | 0.152 | 0.176 |
| Left superior frontal gyrus – Right operculum inferior frontal gyrus | 0.160 | 0.153 |
| Left precuneus – Left calcarine sulcus | 0.118 | 0.296 |
| Right Cuneus – Left Calcarine sulcus | 0.243 | 0.029 |
| Left middle cingulate gyrus – Left triangularis inferior frontal gyrus | 0.109 | 0.332 |
| Right putamen – Right insular cortex | 0.160 | 0.153 |
| Left heschl’s gyrus – Right heschl’s gyrus | -0.019 | 0.866 |
| Left heschl’s gyrus – Right superior temporal gyrus | 0.038 | 0.733 |
| Negative symptoms total |  |  |
| Right posterior cingulate gyrus – Left triangularis inferior frontal gyrus | 0.101 | 0.370 |
| Left orbito middle frontal gyrus – Right orbito inferior frontal gyrus | -0.032 | 0.777 |
| Left orbito middle frontal gyrus – Right operculum inferior frontal gyrus | 0.201 | 0.073 |
| Left orbito middle frontal gyrus – Left orbito inferior frontal gyrus | -0.096 | 0.393 |
| Right orbito middle frontal gyrus – Right triangularis inferior frontal gyrus | -0.075 | 0.507 |
| Right orbito middle frontal gyrus – Left triangularis inferior frontal gyrus | -0.055 | 0.623 |
| Left anterior cingulate gyrus – Right triangularis inferior frontal gyrus | 0.007 | 0.954 |
| Left anterior cingulate gyrus – Left triangularis inferior frontal gyrus | -0.070 | 0.535 |
| Left anterior cingulate gyrus – Left operculum inferior frontal gyrus | 0.078 | 0.491 |
| Right anterior cingulate gyrus – Left orbito inferior frontal gyrus | 0.220 | 0.049 |
| Left superior frontal gyrus – Right operculum inferior frontal gyrus | 0.054 | 0.630 |
| Left precuneus – Left calcarine sulcus | 0.055 | 0.624 |
| Right Cuneus – Left Calcarine sulcus | 0.142 | 0.206 |
| Left middle cingulate gyrus – Left triangularis inferior frontal gyrus | 0.120 | 0.287 |
| Right putamen – Right insular cortex | 0.092 | 0.415 |
| Left heschl’s gyrus – Right heschl’s gyrus | -0.104 | 0.358 |
| Left heschl’s gyrus – Right superior temporal gyrus | -0.040 | 0.726 |
| General psychopathology total |  |  |
| Right posterior cingulate gyrus – Left triangularis inferior frontal gyrus | -0.022 | 0.844 |
| Left orbito middle frontal gyrus – Right orbito inferior frontal gyrus | 0.111 | 0.324 |
| Left orbito middle frontal gyrus – Right operculum inferior frontal gyrus | 0.159 | 0.156 |
| Left orbito middle frontal gyrus – Left orbito inferior frontal gyrus | 0.068 | 0.544 |
| Right orbito middle frontal gyrus – Right triangularis inferior frontal gyrus | -0.029 | 0.795 |
| Right orbito middle frontal gyrus – Left triangularis inferior frontal gyrus | -0.046 | 0.684 |
| Left anterior cingulate gyrus – Right triangularis inferior frontal gyrus | 0.050 | 0.658 |
| Left anterior cingulate gyrus – Left triangularis inferior frontal gyrus | 0.043 | 0.705 |
| Left anterior cingulate gyrus – Left operculum inferior frontal gyrus | 0.100 | 0.373 |
| Right anterior cingulate gyrus – Left orbito inferior frontal gyrus | 0.074 | 0.512 |
| Left superior frontal gyrus – Right operculum inferior frontal gyrus | 0.109 | 0.335 |
| Left precuneus – Left calcarine sulcus | 0.207 | 0.063 |
| Right Cuneus – Left Calcarine sulcus | 0.235 | 0.035 |
| Left middle cingulate gyrus – Left triangularis inferior frontal gyrus | 0.149 | 0.184 |
| Right putamen – Right insular cortex | 0.102 | 0.367 |
| Left heschl’s gyrus – Right heschl’s gyrus | -0.010 | 0.926 |
| Left heschl’s gyrus – Right superior temporal gyrus | 0.059 | 0.599 |
| PANSS total |  |  |
| Right posterior cingulate gyrus – Left triangularis inferior frontal gyrus | -0.007 | 0.953 |
| Left orbito middle frontal gyrus – Right orbito inferior frontal gyrus | 0.100 | 0.376 |
| Left orbito middle frontal gyrus – Right operculum inferior frontal gyrus | 0.197 | 0.078 |
| Left orbito middle frontal gyrus – Left orbito inferior frontal gyrus | 0.071 | 0.528 |
| Right orbito middle frontal gyrus – Right triangularis inferior frontal gyrus | -0.044 | 0.698 |
| Right orbito middle frontal gyrus – Left triangularis inferior frontal gyrus | 0.002 | 0.988 |
| Left anterior cingulate gyrus – Right triangularis inferior frontal gyrus | 0.097 | 0.388 |
| Left anterior cingulate gyrus – Left triangularis inferior frontal gyrus | 0.086 | 0.447 |
| Left anterior cingulate gyrus – Left operculum inferior frontal gyrus | 0.124 | 0.269 |
| Right anterior cingulate gyrus – Left orbito inferior frontal gyrus | 0.165 | 0.141 |
| Left superior frontal gyrus – Right operculum inferior frontal gyrus | 0.130 | 0.246 |
| Left precuneus – Left calcarine sulcus | 0.162 | 0.149 |
| Right Cuneus – Left Calcarine sulcus | 0.251 | 0.024 |
| Left middle cingulate gyrus – Left triangularis inferior frontal gyrus | 0.152 | 0.176 |
| Right putamen – Right insular cortex | 0.139 | 0.215 |
| Left heschl’s gyrus – Right heschl’s gyrus | -0.046 | 0.682 |
| Left heschl’s gyrus – Right superior temporal gyrus | 0.030 | 0.791 |

PANSS; Positive and Negative Syndrome Score.

**References**

1. Hu, J., Shen, L., Sun, G., 2018. Squeeze-and-excitation networks, Proceedings of the IEEE conference on computer vision and pattern recognition, pp. 7132-7141. https://openaccess.thecvf.com/content_cvpr

_2018/html/Hu_Squeeze-and-Excitation_Networks_CVPR_2018_paper\.

1. Kawahara, J., Brown, C.J., Miller, S.P., Booth, B.G., Chau, V., Grunau, R.E., Zwicker, J.G., Hamarneh, G., 2017. BrainNetCNN: Convolutional neural networks for brain networks; towards predicting neurodevelopment. Neuroimage 146, 1038-1049. https://www.sciencedirect.com/science/ article/abs/ pi

i/S1053811916305237.
